# Evaluation of Actin-1 Expression in Wild Caught *Wuchereria bancrofti*-Infected Mosquito Vectors

**DOI:** 10.1155/2020/7912042

**Published:** 2020-05-04

**Authors:** Moses Edache Entonu, Aliyu Muhammad, Iliya S. Ndams

**Affiliations:** ^1^Department of Biochemistry, Ahmadu Bello University, Zaria, Kaduna State, Nigeria; ^2^Africa Centre of Excellence for Neglected Tropical Diseases and Forensic Biotechnology, Ahmadu Bello University, Zaria, Nigeria; ^3^Department of Zoology, Ahmadu Bello University, Zaria, Kaduna State, Nigeria

## Abstract

**Background:**

*Wuchereria bancrofti* is the major cause of lymphatic filariasis transmitted by mosquito vectors. In the vector-parasite interaction and among other proteins, actin-1 has been implicated for successful transmission of the pathogen in laboratory-controlled experiments. However, validation of this finding from the pathogen's natural environment is required.

**Objective:**

This study is aimed at evaluating actin-1 expression upon *Wuchereria bancrofti* infection in mosquito vectors collected during an epidemiology study in Tsafe Local Government Area of Zamfara State, Nigeria.

**Methods:**

Mosquitoes were collected and identified using morphological keys, which include length of maxillary palps, pale spots on the wings, and scale patterns on the abdomen. This was followed by detection of the 188 bp *SspI* marker of *Wuchereria bancrofti* infection using polymerase chain reaction (PCR). The mRNA levels of the *actin-1* gene were evaluated in the infected *Anopheles gambiae sl* and *Culex quinquefasciatus* and their controls, which were adult reared from the larvae in the study area.

**Results:**

The mosquitoes were identified to be *Anopheles gambiae sl* and *Culex quinquefasciatus*, while infection by *Wuchereria bancrofti* was confirmed by amplification of the 188 bp *SspI* marker. A 4.85 and 4.09 relative fold increase in *actin-1* gene expression in *Wuchereria bancrofti*-infected *Anopheles gambiae sl* and *Culex quinquefasciatus* was observed. Thus, for the first time we reported that the *actin-1* gene in wild caught mosquito vectors (*Anopheles gambiae sl* and *Culex quinquefasciatus*) infected with *Wuchereria bancrofti* is upregulated.

**Conclusion:**

The *actin-1* gene is upregulated and similarly expressed during *W. bancrofti* infection in mosquito vectors in the study area and this may likely serve as a biomarker and viable strategy for the control of parasite transmission in endemic areas.

## 1. Introduction

Lymphatic filariasis is a neglected tropical disease (NTD) implicated in the blocking of the lymphatic system caused by parasitic helminths [1]. There are about 500 species of filariae known to infect vertebrates, out of which only eight main species infect humans. These include *Wuchereria bancrofti*, *Brugia malayi*, *Brugia timori*, *Onchocerca volvulus*, *Loa loa*, *Mansonella perstans*, *Mansonella streptocerca*, and *Mansonella ozzardi*. The preferred predilection sites for these parasites are the lymphatic vessels and lymph nodes where they induce development of disfiguring and debilitating clinical symptoms [2]. It has been reported that 886 million people of different countries are living within areas that require preventive chemotherapy to stop the spread of infection. Approximately 80% of these people are living in the following 10 countries: Angola, Cameroon, Côte d'Ivoire, Democratic Republic of the Congo, India, Indonesia, Mozambique, Myanmar, the United Republic of Tanzania, and Nigeria [3]. Fascinatingly, the burden of lymphatic filariasis in Nigeria is predominantly caused by *Wuchereria bancrofti* [4].

The geographical locations and habitat types are major influences where mosquito species function as vectors in any particular endemic area [5]. Current research studies are now focusing on expanding new strategies to abate vector-mediated pathogen transmission which has extended gradually to increasing priority for research on the mosquito-filarial system. However, the success of mosquito-borne disease control is often challenged by reintroduction and resurgence emanating from new or residual human infections that are further disseminated by the vectors, especially when effective human treatment is inadequate. Although the application of insecticides has been largely to reinforce mosquito control for decades, toxicity to humans and emergence of insecticide-resistant traits among mosquito populations have been of great concern, thus reducing the effectiveness of other control measures [6].

Mosquitoes are often exposed to pathogens during feeding through breaks in their cuticle following pathogen-driven cuticular degradation. To resist this, they mount innate cellular and humoral immune responses which sometimes lead to the death of the pathogen through different mechanisms such as lysis, melanization, and hemocyte-mediated phagocytosis [7]. Mosquitoes' immunological, physiological, and structural components can prevent and ameliorate the establishment of filarial parasites [7, 8]. Migratory timing and mechanical crossing of vector midgut barriers [7], as well as ingestion of mitochondria of the thoracic muscle cells of the mosquito, constitute parts of the physical strategies employed by *Wuchereria bancrofti* in order to adapt within the mosquito environment [9, 10]. Epithelial cells of the peritrophic matrix in the mosquito midgut are the first line of defense against many pathogens acquired during blood feeding, and these cells can synthesize several antimicrobial peptides (AMPs). The transcription of innate immunity genes encoding for AMPs is highly dependent on several signalling cascade pathways, including the Janus kinase-signal transducer and activator of transcription (JAK-STAT), toll-like receptor, and immune deficiency pathways [11]. The humoral defense proteins of *Cx. quinquefasciatus* have been elucidated in a laboratory-controlled experiment, during which five proteins were upregulated with a molecular weight of 66, 22, 14, 7, and 40 kDa for transferrin, attacin, lysozyme, defensin, and actin, respectively [12], of which actin which was referred to actin-1 during the primer design [13] is the most critical to parasite infection and development. These proteins are part of the vectors' immune complexes that evolved in response to their interactions with lifecycle stages of pathogens and function in a variety of intracellular processes aimed at ameliorating the cost of vectorial capacity [14]. However, mosquito infected with *Wuchereria bancrofti* from its natural environment has not been examined for these proteins of which actin-1 is the most abundant and often involved in the parasite-vector interaction [15]. Actin-1 is part of the mosquito gut tissue involved in an antagonistic function of limiting parasite infection [16]. Evaluating actin-1 expression in an infected mosquito vector could serve as a possible biomarker for vaccine and drug development in the new strategy for the control of the parasite transmission in endemic areas. This study is aimed at evaluating actin-1 expression in wild caught *Wuchereria bancrofti*-infected mosquito vectors in Tsafe Local Government of Zamfara State, Nigeria.

## 2. Materials and Methods

### 2.1. Chemicals and Reagents

TRIzol reagent, isopropyl alcohol, chloroform, and ethanol (absolute) were obtained from Sigma-Aldrich (USA), RNase-free water, SDS, primers, and agarose powder from Inqaba Biotech South Africa, dNTPs and Taq polymerase from Thermo Fisher Scientific (USA), First-strand cDNA synthesis kit and Luna Universal qPCR master mix from New England BioLab, and ethidium bromide and RNAlater were obtained from Thermo Fisher Scientific (USA). All other reagents were of analytical and molecular grade. Strict adherence to manufacturer's instruction was maintained.

### 2.2. Study Area

The study area was Tsafe Local Government Area in Zamfara State Nigeria with the coordinate 11.57′N and 6.55′E which is about fifty (50) kilometre from Gusau, the state capital. The ethnic groups in the area are predominantly Hausa, Fulani, and Bare-Bare. The major agricultural activity is farming, rearing of animals, and trade [17].

### 2.3. Sample Collection

Convenient method of sampling was used to randomly select three wards from the Local Government Area. In each ward, two sites were selected and five houses were subsequently chosen in which both infected and control mosquitoes were collected by pyrethrum knockdown (PKD) using Baygon as an insecticide of choice. This was used in spraying the indoor-resting mosquitoes between 06:00 am and 10:00 am [3, 18]. The collected mosquitoes were immediately stored in RNAlater at −20°C. 100 mosquito larvae were also collected from the same population to be used as controls. These larvae were identified to be *Anopheles* and *Culex* by the resting position of the larvae which was further confirmed at the emergence of nulliparous using morphological keys; length of maxillary palp, wing spot, mouthpart, and abdominal end.

### 2.4. Morphological Identification of Wild Caught Mosquito Vectors

All the mosquito samples collected were identified by female morphology and male genitalia. Genus level identifications were made with multiple approaches using a morphology key which includes length of maxillary palp, wing spot, mouthpart, and abdominal end at 40× magnification [19].

#### 2.4.1. Extraction of DNA

DNA was extracted using the TRIzol® reagent [20]. The mosquitoes parts (the head, thorax, and abdomen) were homogenized in the TRIzol® reagent using a glass Teflon at 25°C and vortexed to ensure thorough and complete isolation and solubilisation of the macromolecules. The resulting mixture was then separated into three phases (a lower red phenol-chloroform phase, an interphase, and a colourless upper aqueous phase). 250 *μ*l back extraction buffer (4 M guanidine thiocyanate; 50 mM sodium citrate; 1 M Tris, pH 8.0) was added to the phenol phase and the interphase, and the mixtures were incubated at room temperature for 10 min. Samples were then centrifuged at 13200 rpms for 15 minutes at 4°C. The upper phase was removed and an equal volume of 100% isopropanol was added and incubated overnight at −80°C. After incubation, samples were centrifuged at 13,200 rpm for 15 minutes at 4°C. The supernatant was removed, and the pellets were washed 3 times with 70% ethanol and then eluted with the TAE buffer in a final volume of 20 *μ*l at pH 8.0 and the purity was determined at A260/280 [21, 22].

#### 2.4.2. Molecular Identification of *Wuchereria bancrofti*

*Wuchereria bancrofti* collected from the wild caught infected mosquitoes were identified using the PCR technique. Primers (Table 1) were used to amplify the 188 bp *SspI* repeats of *Wuchereria bancrofti* [23]. PCR was performed in a thermocycler (Applied Biosystems) with conditions set at 96°C for 15 seconds followed by 25 cycles of 94°C for 3 seconds, 56°C for 3 seconds, and 72°C for 5 seconds [24, 25]. A total reaction volume of 25 *μ*l containing 2 *μ*l of DNA sample, 10 mM Tris-HCl, pH 9.2, 2 mM MgCl_2_, 75 mM KCl, 1.2 mM of each deoxynucleotide triphosphate, 10 pmol each of *SspI* forward and reverse oligonucleotide primers (Table 1), and 2 unit of Taq polymerase was used.

*(1) RNA Extraction*. The clear aqueous surface obtained from the mosquito homogenate during DNA isolation was transferred into a fresh 1.5 mL RNase-free tube with equal volume of RNase-free 70%. Ethanol was added after which the mixture was transferred into an RNeasy spin column and centrifuged at 18,000 rpm for 30 seconds. Thereafter, the flow through was discarded. 700 *μ*l of the RW1 buffer was added to the column and centrifuged at 18,000 rpm for 30 seconds and transferred to a new collection tube into which 500 *μ*l of RPE buffer was added and centrifuged for 2 minutes at maximum speed. The flow through was discarded, and the column was transferred to a 1.5 ml RNase-free collection tube and eluted with 20 *μ*l RNase-free water. The concentration and purity of extracted RNA were determined at A260/A280.

*(2) Complimentary DNA (cDNA) Synthesis*. Reverse transcription of the RNA to cDNA was carried out with the SuperScript First-Strand Synthesis System. The procedure was based on Invitrogen's protocol (New England Bio Lab_inc_) in a 20 *μ*l reverse transcription mixture. 1 *μ*l of the isolated RNA was mixed with 2 *μ*l of random hexamers, and nuclease-free water was added to make up the total volume. It was then incubated at 65°C for 5 minutes, and thereafter, ProtoScript II reaction mix (2x) and ProtoScript II enzyme mix (10x) were added and then incubated at 25°C for 5 minutes followed by 42°C for 1 hour. Finally, the enzyme reaction was inactivated at 80°C for 5 minutes.

*(3) Amplification of Actin-1*. Polymerase chain reaction was carried out in a thermal cycler (Applied Biosystems) with a total reaction volume of 50 *μ*l using a universal primer sequence (Table 1) for the amplification of actin-1 cDNA [13]. The PCR mix contained 5 *μ*l 10 X PCR buffer, 1.5 *μ*l Mgcl_2_ (50 mM), 1 *μ*l dNTP (10 mM), 1 *μ*l of each primer (10 mM), 0.4 *μ*l Taq polymerase (5 U/*μ*l), 2 *μ*l cDNA, and 38.1 *μ*l of DEPC-treated water with conditions set at 96°C for 15 seconds followed by 25 cycles of 94°C for 3 seconds, 56°C for 3 seconds, and 72°C for 5 seconds [25].

*(4) Agarose Gel Electrophoresis*. The obtained PCR products from identification of *Wuchereria bancrofti* and actin-1 were visualized on prepared 1.5% agarose gel stained with 0.5 *μ*g/ml ethidium bromide to detect the amplified DNA fragments. Accordingly, 5 *μ*L of each PCR product was added to 1 *μ*L of the loading dye for electrophoresis. The gel was prepared by weighing 1.5 g of agarose powder and dissolved in 100 ml of TAE buffer and poured on a casting tray to allow polymerization and solidification at room temperature and then electrophoresis was performed using 1x TAE in a minigel system at 100 volts for one hour. The gel was visualized on a UV transilluminator where the sizes of the PCR products were estimated by comparison with the mobility of a 100 bp DNA molecular ladder [26].

*(5) Sequencing of Actin-1 Gene Amplicon*. The PCR products were purified using a QIAQUICK PCR Purification Kit (QIAGEN, USA) following the manufacturer's protocol. Sequencing was carried out using the BigDye Terminator v3.1 Cycle Sequencing Kit on purified PCR products in a DNA thermal cycler.

*(6) Quantitative Polymerase Chain Reaction (qPCR)*. Evaluation of mRNA levels was done using the SYBRGreenER™ qPCR SuperMix Universal (Thermo Fisher Scientific, USA) following the manufacturer's instructions, with a total reaction volume of 20 *μ*l. Primers (Table 1) for both actin-1 and GAPDH (internal control) were equally used in triplicates. For the reaction, 2 *μ*l of reverse-transcribed products (cDNA), 10 *μ*l Luna universal qPCR mix, 1 *μ*l of RNAse-free water, and 1 *μ*l each of forward and reverse primers were present in a 20 *μ*l final volume. Thermo cycler conditions consisted of 1 cycle −95°C for 15 minutes and forty cycles 95°C for 15 seconds, 62°C for 15 seconds, and 72°C for 30 seconds. A melting curve was obtained for each quantitative PCR run, and the second derivative maximum method was used to determine the crossing point (Cp) for individual samples [27]. The real-time PCR data generated (Table 2) were analyzed using the 2^−ΔΔCT^ relative quantization method [28].

### 2.5. Data Analysis

To address biological variability, experiments were repeated at least three (3) times and appropriate data were presented as mean ± SEM. Variability between groups was measured using the *t*-test with the aid of Statistical Package for Social Sciences Software version 20.0. Level of significance measured at *P* < 0.05 was considered statistically significant using least significance difference (LSD).

## 3. Results

### 3.1. Mosquito Vector Distribution and Morphological Identification

Table 3 shows that 100 larvae were collected from breeding sites in the study area and reared in the laboratory to adult, of which 50 were *Culex* and 50 *Anopheles* as identified from the larvae stage; however, only 41 and 33 emerged from *Culex* and *Anopheles*, respectively, due to mortality. Out of the 2000 wild caught mosquitoes, 784 were *Anopheles gambiae sl* and 925 were identified to be *Culex quinquefasciatus* of which one from each group was found to be positive with *W. bancrofti*. The remaining 291 wild caught mosquitoes were identified to be *Aedes aegypti* and not infected with *W. bancrofti*. The morphological features used for the identification included length of maxillary palps, wing pale spots, and scale patterns on the abdomen as previously described [29].

### 3.2. Molecular Detection of *Wuchereria bancrofti* and Amplification of Actin-1

**Figure 1:**
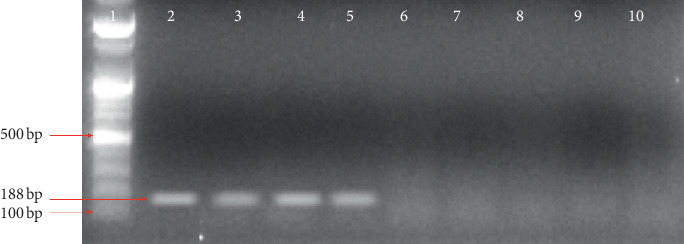
Agarose gel electrophoresis to detect *SspI Wuchereria bancrofti*. Lane 1 = 100 bp molecular marker; Lane 2 and 3 = *Anopheles* (infected with *Wuchereria bancrofti*); Lane 4 and 5 = *Culex* (infected with *Wuchereria bancrofti*); Lane 6 and 7 = *Anopheles* (negative); Lane 8 and 9 = *Culex* (negative).

**Figure 2:**
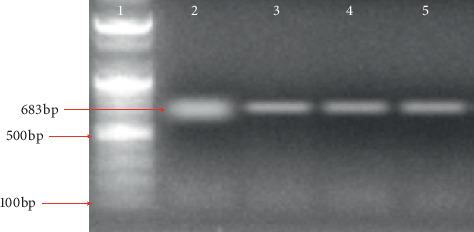
Agarose gel electrophoresis to amplify actin-1 in both infected and control mosquitoes. Lane 1 = 100 bp molecular marker, Lane 2 and 3 = infected mosquito (*Anopheles* and *Culex*, respectively), and Lane 4 and 5 = control (*Anopheles* and *Culex*, respectively).

**Figure 3:**
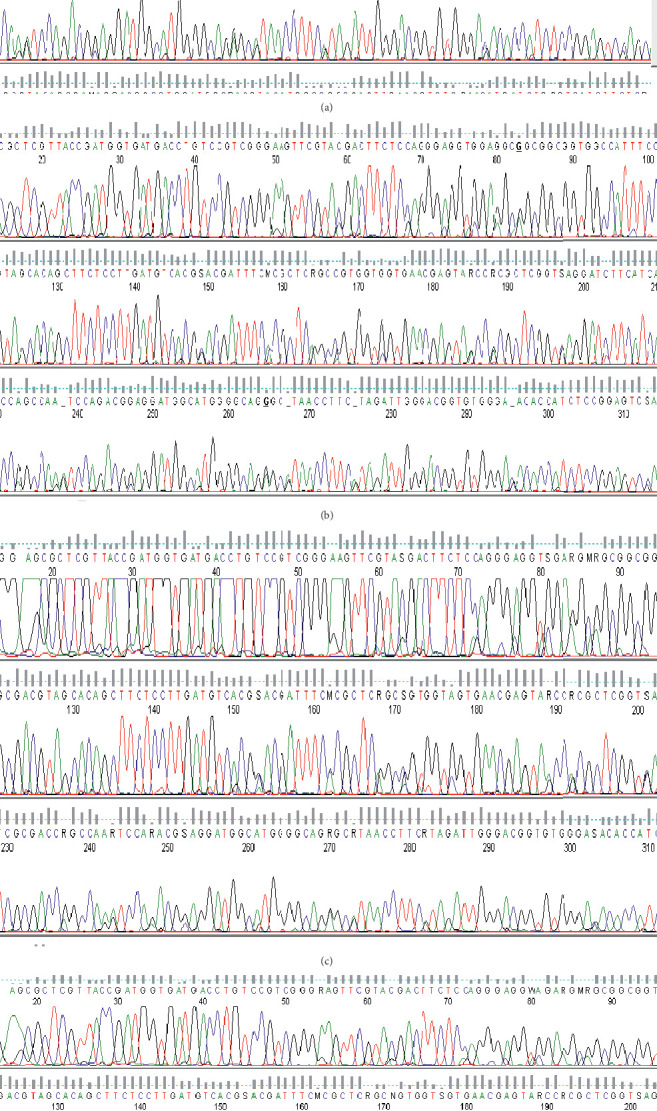
(a) Sequence chromatograms of actin-1 with a region used for primer design from infected *Anopheles gambiae sl*. (b) Sequence chromatograms of actin-1 with a region used for primer design from control *Anopheles gambiae sl*. (c) Sequence chromatograms of actin-1 with a region used for primer design from infected *Culex quinquefasciatus*. (d) Sequence chromatograms of actin-1 with a region used for primer design from control *Culex quinquefasciatus*.

The filarial parasite from this research was identified by polymerase chain reaction using *SspI* primers [23] with an amplicon size of 188 bp as shown in Figure 1.  Figure 2 shows the amplicon of *actin-1* gene amplified using an actin-1 universal primer. The bands from gel were excised (Figure 2), purified, and sequenced (Figures 3(a)–3(d)).

### 3.3. Expression of Actin-1

**Figure 4:**
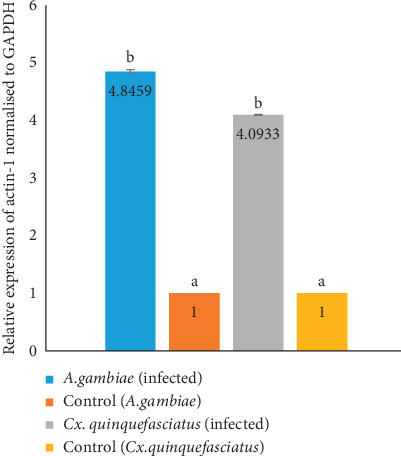
Expression of actin-1 in infected Anopheles gambiae and Culex quinquefasciatus. Bars (means) with different superscript are statistically significant at p < 0.05.

Figure 4 shows the result of actin-1 expression in wild caught *Wuchereria bancrofti*-infected *Anopheles gambiae sl* and *Culex quinquefasciatus*. The expression fold of the *actin-1* gene in *Anopheles gambiae* was increased by 4.85 and that of *Culex quinquefasciatus* showed a fold increase of 4.09 relative to controls. The proportional fold increase of *actin-1* gene expression was found to be higher in *Anopheles gambiae sl* by 0.7526 when compared with *Culex quinquefasciatus* although not statistically significant (*P* > 0.05).

## 4. Discussion

*Wuchereria bancrofti* is the major cause of lymphatic filariasis transmitted by mosquito vectors. In the vector-parasite interaction and among other proteins, actin-1 has been implicated for successful transmission of the pathogen. Actin-1 is an extracellular pathogen recognition factor that mediates antibacterial defense [12]. Insect actin-1 is secreted from cells upon immune challenge through an exosome-independent pathway, and it binds to the surfaces of bacteria, mediating their phagocytosis and direct killing. Globular and filamentous actins display distinct functions as extracellular immune factors [30]. Unfortunately, in an endemic country like Nigeria, there has been little or no attention given to the pathogen-vector interactions in research studies to decipher the biological factors involved. In this communication, we identified and evaluated *actin-1* gene expression in wild caught *Wuchereria bancrofti*-infected mosquito vectors obtained from their natural environment during an epidemiological study in Tsafe Local Government Area of Zamfara State, Nigeria.

From this study, only 2 mosquitoes were found to be infected with a filarial nematode from which one was *Anopheles gambiae sl* and the other was *Culex quinquefasciatus*. The filarial specimen was *Wuchereria bancrofti* confirmed by PCR amplification of the *SspI* gene for the detection of *Wuchereria bancrofti* with a corresponding amplicon size of 188 bp. This results corroborate with the result which tested the hypothesis that *Wuchereria bancrofti* can be identified in mosquito vectors using the *SspI* repeats [24]. Similarly, a survey of bancroftian filariasis infection in humans and *Culex* mosquitoes in the western Brazilian Amazon region showed that implications for transmission and control using *SspI* repeats as a signature to the parasite have proven to be a powerful tool to evaluate the contamination intensity of the mosquitoes in endemic areas. Due to these advantages, it has replaced the conventional dissection methods for the diagnosis [31]. Similarly, the *SspI* repeat has a unique recognition site for restriction endonuclease in all or most of the repeat copies. This DNA family is dispersed, genus-specific, and exists in all of the different geographic isolates of *Wuchereria bancrofti* tested [23].

The expression fold increase of actin-1 in *Anopheles gambiae* was found to be 4.85 and 4.09 for *Culex quinquefasciatus* as compared to the controls. These values indicate that the expression of actin-1 is upregulated in *Culex quinquefasciatus* which agrees with other reported upregulation of actin-1 protein upon infection and development of the filarial parasites within their vectors [12]. Actin-1 expression in *Anopheles gambiae* was 0.7526 more than that of *Culex quinquefasciatus* which may be as a result of the fact that *Anopheles* species are the predominant vectors in the study area and they have adapted to ameliorating the effect of parasite infection via various mechanisms which include melanisation, encapsulation, and phagocytosis. Mosquito immune machinery involves biochemical reactions using exoskeleton cuticle, epidermis physical and chemical properties, gut epithelium, and reproductive accessory glands [32]. The major feature in this immune machinery is the recognition of nonself-parts via pattern recognition receptors (PRRs) that identify pathogen-associated molecular patterns (PAMPs) which trigger the activation of cellular and humoral immune responses, both acting together to eliminate pathogens by surrounding of pathogens using melanin [33]. Typically, cellular responses include phagocytosis, nodulation, and encapsulation, while humoral include antimicrobial systemic molecules, nitric oxide (NO) production, and lysozymes [14]. Mosquitoes also possess innate immunity to combat pathogen infection [11], supporting the facts that actin-1 is indeed part of the innate immune response as a consequence of parasite-vector interactions [12]. The innate immune system includes phagocytic cells in the circulation and tissues. These cells recognize and engulf pathogens through germline-encoded pattern recognition receptors specific for microbial products such as the toll-like receptors [34]. The toll pathway in mosquitoes has been found to control hemocyte proliferation and plays a critical role in cellular immune response, for the expression of actin-1, transferrin, and defensin proteins critical to encapsulation and killing of parasites [35].

## 5. Conclusion

Wild caught *Anopheles gambiae sl* and *Culex quinquefasciatus* infected with *W. bancrofti* from Tsafe Local Government Area of Zamfara State Nigeria showed upregulation of the *actin-1* gene. Therefore, targeting actin-1 as one of the protein that is upregulated due to interaction between the parasite and the vector could serve as a new strategy for the control of the parasite transmission in endemic areas.

## Figures and Tables

**Table 1:** List of primers used for conventional polymerase chain reaction (PCR) and relative quantitative real-time polymerase chain reaction (qPCR).

Genes	Amplicon size (bp)	Forward 5′ ⟶ 3′	Reverse 5′ ⟶ 3′
*SspI*	188	CGTGATGGCATCAAAGTAGGG	CCCTCACTTACCATAAGACAAC
*Actin-1* (PCR)	683	ATGGTCGGCATGGGTCAGAAGGACTC	GATTCCATACCCAGGAAGGATGG
*Actin-1* (qPCR)	154	GCACGGTATCATCACCAACTG	CATGATCTGGGTCATCTTCTCG
*GAPDH*	194	ACAGACGCTAGTTATCAACGTA	ACCGTGGGTCGAATCGTA

**Table 2:** Cycle threshold (Ct) values and calculations for relative fold change in the expression of the *actin-1* gene.

Samples	Ct test	Mean Ct (test)	Ct ref (GAPDH)	Mean Ct (ref)	∆Ct test (mean Ct of test‐ mean Ct ref)	∆∆Ct (∆Ct test‐∆Ct control)	2^−∆∆Ct^	SD	SEM
*Infected A*	15.87		18.93						
	15.80	15.81	18.86	18.87	−3.06				
	15.77		18.82						
						−2.28	4.85	0.0573	0.033

*Control A*	16.33		17.12						
	16.28	16.25	17.08	17.04	−0.79				
	16.13		16.91						

*Infected B*	15.6		18.47						
	15.68	15.67	18.55	18.60	−2.87				
	15.74		18.6						
						−2.03	4.09	0.0132	0.008

*Control B*	15.78		16.62						
	15.85	15.82	16.68	16.65	−0.84				
	15.82		16.66						

A: *Anopheles gambiae* and B: *Culex quinquefasciatus*.

**Table 3:** Distribution of mosquito vectors and the infection rate of *Wuchereria bancrofti*.

	Total samples	Infected mosquitoes	Mosquito types	
			*Culex quinquefasciatus*	*Anopheles gambiae*
Wild caught	2000	2 (0.002%)	924 (46.2%)	783 (39.2%)
Laboratory bred	100	—	41 (82.0%)	33 (66.0%)

## Data Availability

The raw data and samples used to support the findings of this study are available from the corresponding author upon request.
